# Effect of a Physiotherapist-Guided Home-Based Exercise Intervention on Physical Capacity and Patient-Reported Outcomes Among Patients With Acute Pulmonary Embolism: A Randomized Clinical Trial

**DOI:** 10.1001/jamanetworkopen.2020.0064

**Published:** 2020-02-28

**Authors:** Nanna Rolving, Barbara C. Brocki, Jannie R. Bloch-Nielsen, Torben B. Larsen, Frank L. Jensen, Hanne R. Mikkelsen, Pernille Ravn, Lars Frost

**Affiliations:** 1 Diagnostic Center, Silkeborg Regional Hospital, Silkeborg, Denmark; 2 Department of Occupational Therapy and Physiotherapy, Aalborg University Hospital, Aalborg, Denmark; 3 Thrombosis and Drug Research Unit, Department of Cardiology, Aalborg University Hospital, Aalborg, Denmark; 4 Department of Physical and Occupational Therapy, Regional Hospital Herning, Herning, Denmark; 5 Diagnostic Center, Department of Cardiology, Silkeborg Regional Hospital, Silkeborg, Denmark; 6 Department of Clinical Medicine, Aarhus University, Aarhus, Denmark

## Abstract

**Importance:**

Survivors of acute pulmonary embolism (PE) experience long-term negative physical and mental consequences, but the effects of rehabilitation on outcomes among these patients have not been investigated.

**Objective:**

To investigate the effect of a rehabilitation intervention, comprising an 8-week home-based exercise program and nurse consultations, on physical capacity and patient-reported outcomes among patients with acute PE.

**Design, Setting, and Participants:**

This multicenter randomized clinical superiority trial was conducted at 4 regional hospitals and 1 university hospital in Denmark. The 140 consecutively included participants had been diagnosed with an acute PE between April 2016 and February 2018 and had 6 months of follow-up. An intention-to-treat analysis was conducted.

**Intervention:**

Patients in the control group received a brief nurse consultation, while patients in the exercise group participated in an 8-week home-based exercise program in addition to receiving nurse consultations.

**Main Outcomes and Measures:**

The primary outcome was the Incremental Shuttle Walk Test, and secondary outcomes were the Pulmonary Embolism Quality of Life and the EuroQol–5 Dimensions–3 Levels questionnaires, self-reported number of sick-leave days, and self-reported use of psychotropic drugs.

**Results:**

A total of 140 patients (90 [64.3%] men) were included, with a mean (SD) age of 61 (11) years. Of 70 participants (50.0%) randomized to each group, 69 participants (49.3%) received the intervention and 68 (48.6%) received the control intervention. Both groups achieved improvements in all outcomes (eg, mean [SD] improvement on Incremental Shuttle Walk Test: control group, 78 (127) m; intervention group, 104 [106] m; median [interquartile range] improvement on Pulmonary Embolism Quality of Life: control group, −17 [−22 to −11] points; intervention group, −20 [−24 to −15] points). Between-group differences were nonsignificant. The mean differences between the intervention group and the control group were 25 m (95% CI, −20 to 70 m; *P* = .27) on the Incremental Shuttle Walk Test, 3.0 points (95% CI, −3.7 to 9.9 points; *P* = .39) on the Pulmonary Embolism Quality of Life questionnaire, and 0.017 point (95% CI, −0.032 to 0.065 point; *P* = .50) on the EuroQol–5 Dimensions–3 Levels questionnaire. Of the 27 patients in the intervention group on sick leave at baseline, 24 (88.9%) reported fit-for-duty at the 6-month follow-up, and of 18 patients in the control group on sick leave, 17 (94.4%) reported fit-for-duty at the 6-month follow up. The between-group risk difference was not significant (5.5 points; *P* = .49).

**Conclusions and Relevance:**

An 8-week rehabilitation intervention with exercise added to nurse consultations did not show significantly better outcomes than nurse consultations alone. However, because of a ceiling effect on the primary outcome of physical capacity and an inclusion of patients with a low comorbidity burden and low PE disease severity, definitive conclusions could not be drawn. Initiating an exercise intervention shortly after pulmonary embolism was safe and without adverse events.

**Trial Registration:**

ClinicalTrials.gov Identifier: NCT02684721

## Key Points

**Question:**

Can a rehabilitation intervention consisting of physiotherapist-guided home-based exercise intervention and nurse consultations improve physical capacity and quality of life among patients with acute pulmonary embolism more than nurse consultations alone?

**Findings:**

In this randomized clinical trial of 140 participants, improvements in physical capacity and quality of life were achieved with no differences between the intervention and control groups. No adverse events were reported.

**Meaning:**

Adding an exercise intervention to nurse consultations did not increase physical capacity or quality of life, but the study findings were limited by a highly selected group of patients with a low burden of comorbidity, exhibiting ceiling effects on the physical capacity measure.

## Teaser

This randomized clinical trial investigates the effect of a rehabilitation intervention, comprising an 8-week home-based exercise program and nurse consultations, on physical capacity and patient-reported outcomes among patients with acute pulmonary embolism.

## Introduction

Pulmonary embolism (PE) is a serious condition leading to the hospitalization or death of more than 500 000 individuals annually in Europe, the United States, and Canada.^1,2^ While developments in early diagnosis and treatment of acute PE have resulted in improved survival rates,^3,4^ a number of studies indicate long-term impairments in physical and mental well-being among survivors of PE,^5,6,7,8,9,10,11,12,13,14^ similar to those reported for patients with other life-threatening diseases.^15,16^ The value of rehabilitation after an acute PE to counteract these negative consequences has not yet been established, given that rehabilitation is not currently part of standard care following PE.^4^ Only a few studies have investigated the effect and safety of physical activity or exercise following PE. Two observational studies^17,18^ have reported no adverse events related to a 3-week inpatient rehabilitation program. To our knowledge, only 1 randomized clinical trial^19^ has examined the effects of an exercise program, compared with telephone contact, on body mass index and cardiorespiratory fitness (ie, VO_2peak_). No definitive conclusions could be drawn from this study because only 19 patients were included, of whom just 9 had PE.^19^ No patient-reported outcome measures were reported.^19^ Looking to studies of rehabilitation for cardiac disease or chronic obstructive pulmonary disease, there is well-founded evidence that physical rehabilitation has a positive effect on quality of life, physical capacity, fatigue, and dyspnea.^20,21^

The aim of our study was to investigate the effect of a rehabilitation intervention, comprising a physiotherapist-guided 8-week home-based exercise program in addition to nurse consultations, on physical capacity and patient-reported outcomes among patients with acute PE. We hypothesized that participating in the exercise program would lead to significantly larger improvements in physical capacity and patient-reported outcomes compared with an active control intervention with nurse consultations only.

## Methods

All patients were informed both in writing and verbally about the purpose and details of the study. Written consent was completed at first visit (ie, 2-3 weeks after discharge), before the baseline test. The study was conducted in agreement with the Helsinki Declaration and was approved by the ethics committee of Central Denmark Region. The study was reported according to the Consolidated Standards of Reporting Trials (CONSORT) reporting guideline for nonpharmacologic randomized trials.^22^

The study was designed as a multicenter randomized clinical superiority trial, comparing the effect of nurse consultations combined with physiotherapist-guided 8-week home-based exercise program vs nurse consultations alone using a 1:1 allocation ratio. Eligible patients were recruited from 4 regional hospitals and 1 university hospital in Denmark between April 2016 and February 2018.

### Participants

Patients were eligible if they presented with an objectively verified first-time acute PE, received anticoagulant drugs, were aged 18 to 80 years, and were competent in the Danish language. Patients were excluded in cases of severe comorbidity (eg, severe heart disease or chronic obstructive pulmonary disease, active cancer, severe psychiatric disease), inability to perform the Incremental Shuttle Walk Test (ISWT), or pregnancy.

The simplified PE severity index (PESI) was estimated at the time of presentation with PE (simplified PESI score: 1 point if cancer, 1 point if chronic obstructive pulmonary disease, 1 point if pulse rate greater than 110 bpm, 1 point if systolic blood pressure less than 100 mm Hg, 1 point if arterial oxyhemoglobin saturation less than 90%, 1 point if older than 80 years).^23^ The age criteria was expanded from 18 to 70 years to 18 to 80 years shortly after the study was initiated because it became evident that many eligible patients would be excluded because of the upper age limit. Physiotherapists or nurses in the wards provided eligible patients with written and verbal information about the study during hospitalization. If required, patients were given 2 days to consider participation, and if they agreed, they were contacted by telephone after discharge.

### Randomization

The first nurse consultation and baseline measurements took place 2 to 3 weeks after discharge from the hospital. Following the baseline test, patients were randomly allocated to either the exercise group (EG) or the control group (CG) using opaque, sealed envelopes. The allocation sequence, using block randomization (by hospital), was generated by the primary investigator (N.R.), who was not otherwise involved in the practical enrollment and assignment of patients to the 2 groups. The enrollment and randomization procedure was handled by the physiotherapists (J.R.B.-N. and F.L.J., among others) at the different hospitals from which patients were discharged. The physiotherapists were provided with blocks of 6 envelopes, and when 2 envelopes remained in 1 block, these were included in a new block of 6 envelopes to ensure allocation concealment.

### Masking

Owing to the nature of the intervention, the patients and the physiotherapists providing the intervention could not be masked to group allocation. The physiotherapists performing the follow-up tests were masked to group allocation. Nurses and physicians at the departments, including nurses providing consultations to both groups, were similarly masked for group allocation.

### Interventions

**Figure 1.**
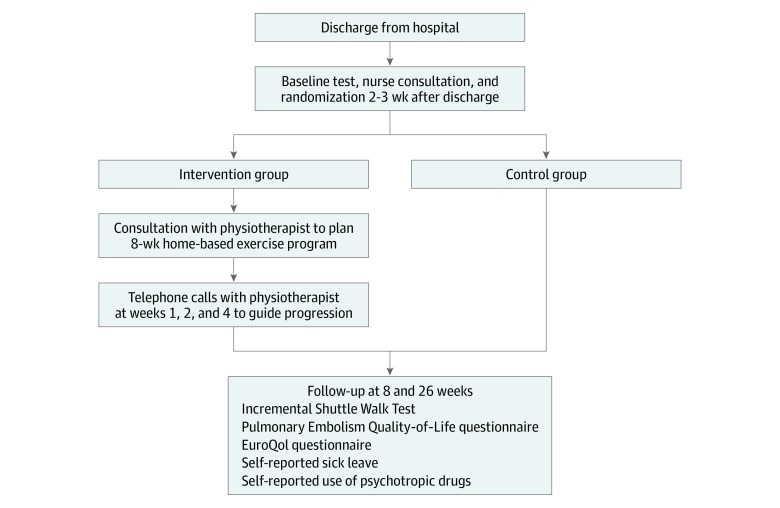
Overview of Trial Design Initial nurse specialist consultation, which took place 2 to 3 weeks after discharge from the hospital, included control of anticoagulation treatment, control of renal function, patient education, distribution of compression socks, and instructions for using a telephone hotline. Both groups were allowed to contact the nurse specialist at the hospital at any time during the 6 month project period.

A detailed description of the interventions is provided in the previously published study protocol^24^ and appears in Supplement 1. An overview of the study design and intervention groups is provided in Figure 1.

### Control Group

During hospitalization, all patients received anticoagulant treatment as well as information and advice about PE. During the 6 months following discharge, patients in the CG received 1 or more consultations with a specialized nurse, depending on the patient’s needs.

### Exercise Group

In addition to nurse consultations, patients in the EG participated in a physiotherapist-guided 8-week home-based exercise program, initiated by a 1-hour consultation with a physiotherapist. The physiotherapist recommended that patients exercise a minimum of 3 times per week for 30 to 60 minutes, including several intervals of high intensity exercise during each session. Patients were encouraged to choose an exercise modality they enjoyed or were familiar with to maximize compliance with the program. Progression was guided by the physiotherapist via follow-up telephone calls after 1, 2, and 4 weeks. Patients filled out an exercise diary with type, frequency, and intensity of training sessions during the 8 weeks. Compliance was defined as completing at least 75% of planned exercise sessions, ie, a minimum of 18 of 24 sessions.

### Outcome Measures

All outcomes were measured as change from baseline (ie, 2-3 weeks after hospital discharge) to follow-up after 2 and 6 months. The primary outcome was physical capacity using the ISWT. The secondary outcomes were quality of life using the Pulmonary Embolism Quality of Life (PEmbQoL) questionnaire and the EuroQol–5 Dimensions–3 Levels (EQ-5D-3L) questionnaire, sick leave (self-report), and use of psychotropic drugs (self-report).

The ISWT is considered a valid and reliable measure in patients with heart and lung diseases.^25^ When performing the ISWT, the patient walks on a 10-m track with auditory beeps indicating walking speed. The speed increases every minute until running speed and ends when the patient cannot keep up with the beeps. The total number of meters walked is recorded.^25^ Patients rated their dyspnea before and immediately after the test using the Borg Category-Ratio 10 scale^26^ and were instructed to keep walking and/or running until they reached at least a level of 7 on the scale.

The PEmbQoL is a disease-specific quality of life questionnaire,^27^ consisting of 40 questions regarding frequency of complaints, limitations in activities of daily living, work-related problems, social limitations, intensity of complaints, and emotional complaints. A total score is calculated by adding the scores of each dimension and transforming them from a scale of 6 to 27 to a scale of 0 to 100.^28^ Higher scores indicate a worse outcome, and a minimal clinically important change of 15 points has been determined.^28^ The PEmbQoL has been validated in a Scandinavian setting.^27^

The EQ-5D-3L is a generic quality of life questionnaire comprising 5 dimensions (ie, mobility, self-care, usual activities, pain or discomfort, and anxiety or depression). Each dimension has 3 levels (ie, no problems, some problems, and extreme problems). The scores fall on a scale of −0.624 to 1.000 (perfect health) and has been validated in Danish settings, including the development of Danish preference values.^29,30^

Self-reported sick leave was reported by stating the number of sick leave days during the previous 4 weeks according to the following categories: 0 days; 1 to 4 days per week; 5 to 7 days per week. For this analysis, only nonretired patients were included.

Use of psychotropic drugs was assessed by stating the average number of days of use per week within the previous 4 weeks according to the following categories: 0 days per week, 1 to 4 days per week, and 5 to 7 days per week. Baseline data, including demographic and clinical data, were retrieved from the patients’ medical records.

### Sample Size Calculation

A minimum clinically relevant difference for the ISWT has not been established for a PE population. Therefore, we used values from a cardiac population, set to 70 m, with an SD of 139 m.^31^ A sample size of 62 patients in each group was required at a significance level of 5% and a power of 80%. Assuming an 85% retention rate (taking into consideration attrition because of loss to follow up), a sample of 142 patients was needed.

### Statistical Analysis

Data were entered into a database using the research electronic data capture (REDCap; Vanderbilt University) tool.^32^ Data entry was performed twice for quality assurance by 2 of us (J.R.B.-N. and P.R.). All data management and analysis was performed by the first author (N.R.). The data distribution and choice of analysis was additionally assessed and approved by an independent statistician who was not otherwise involved in the study. Baseline demographic variables were described using means and SDs or medians and interquartile ranges (IQRs) for continuous variables and counts and percentages for categorical variables. Within-group changes in the ISWT, PEmbQoL, and EQ-5D-3L were compared using a paired *t* test or Wilcoxon signed rank test depending on the data distribution. Student *t* test or Wilcoxon rank sum test were applied for assessment of between-group differences. Because of the categorical distribution, sick leave and use of psychotropic drugs were analyzed using a χ^2^ test. Analysis of data was performed according to intention-to-treat principles. Furthermore, a complementary as-treated analysis was performed for patients complying with a minimum of 75% of the exercise sessions to evaluate the efficacy of the intervention. A sensitivity analysis was performed for the primary outcome (ie, ISWT) to assess the robustness of our finding, using the last observation carried forward method. Data analysis was performed with Stata version 15.0 (StataCorp). Statistical significance was set at *P* < .05, and all tests were 2-tailed.

## Results

### Patient Flow and Baseline Characteristics

**Figure 2.**
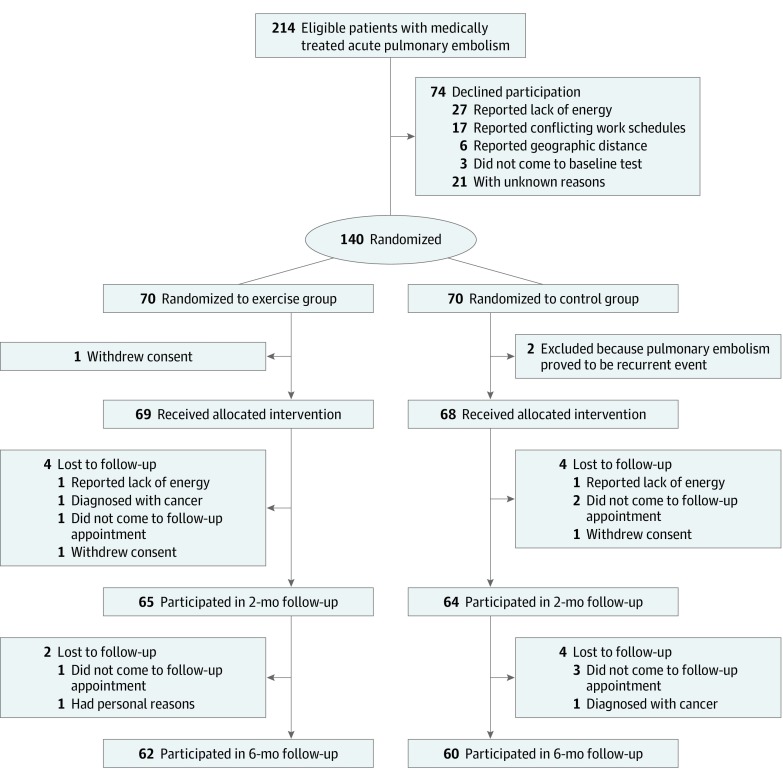
Study Flow Chart

During the study period, 214 eligible patients were approached; 140 (65.4%) accepted participation and were included in the study. Figure 2 outlines the flow of patients through the trial, including reasons for declining participation and dropout at the 2-month and 6-month follow-ups.

The 140 included patients had a mean (SD) age of 61 (11) years of age, and most (90 [64.3%]) were men. Overall, 70 participants (50.0%) were randomized to each group. In the EG, 1 participant (1.4%) withdrew consent, and in the CG, 2 participants (2.8%) were excluded because PE proved to be recurrent, resulting in 69 participants (49.3%) in the EG and 68 (48.6%) in the CG. These participants were generally low-risk PE patients, in terms of simplified PESI score (0: 47 [68.5%] in EG and 50 [71.5%] in CG) and right ventricle load (EG: 25 [36.2%]; CG: 27 [39.7%]). The patients’ baseline characteristics appear in Table 1. Overall, the 2 groups were balanced on all demographic and baseline outcome variables.

**Table 1.** Baseline Characteristics

Characteristic	Group, No. (%)	
	Exercise (n = 69)	Control (n = 68)
Men	42 (60.9)	48 (70.6)
Age, mean (SD), y	60.3 (12.2)	61.8 (10.5)
BMI, mean (SD)	28.7 (6.1)	29.6 (5.6)
PE type		
Central	40 (58.0)	30 (44.1)
Peripheral	14 (20.3)	22 (32.4)
Central and peripheral	15 (21.7)	16 (23.5)
Simplified PE Severity Index score		
0	47 (68.1)	50 (73.5)
1	14 (20.3)	15 (22.1)
2	7 (10.1)	5 (7.4)
3	1 (1.4)	0
Right ventricular strain at index event^a^	25 (36.2)	27 (39.7)
Medication		
NOAC	56 (81.2)	57 (83.8)
Warfarin	13 (18.8)	11 (16.2)
Length of hospitalization, d		
Mean (SD)	5.5 (3.2)	5.6 (3.3)
Median (range)	5 (1-16)	5 (1-16)
Employment status		
Employed	31 (44.9)	28 (41.2)
Unemployed	3 (4.3)	1 (1.5)
Retired	32 (46.4)	36 (52.9)
Other	3 (4.3)	3 (4.4)
Marital situation		
Living with spouse	59 (85.5)	47 (69.1)
Living alone	10 (14.5)	20 (29.4)
Other	0	1 (1.5)
Smoking status		
Never	32 (46.4)	29 (42.6)
Previous	33 (47.8)	34 (50.0)
Smoker	3 (4.3)	5 (7.4)
Missing	1 (1.4)	2 (2.9)
Average level of physical activity		
Very high	4 (5.8)	3 (4.4)
High	20 (29.0)	18 (26.5)
Moderate	38 (55.1)	45 (66.2)
Low	6 (8.7)	2 (2.9)

Abbreviations: BMI, body mass index (calculated as weight in kilograms divided by height in meters squared); NOAC, novel oral anticoagulant; PE, pulmonary embolism.

^a^ Right ventricle strain indicates right ventricle dilatation, D-shape, and/or tricuspid regurgitant gradient of at least 40 mm Hg.

### Outcomes

**Figure 3.**
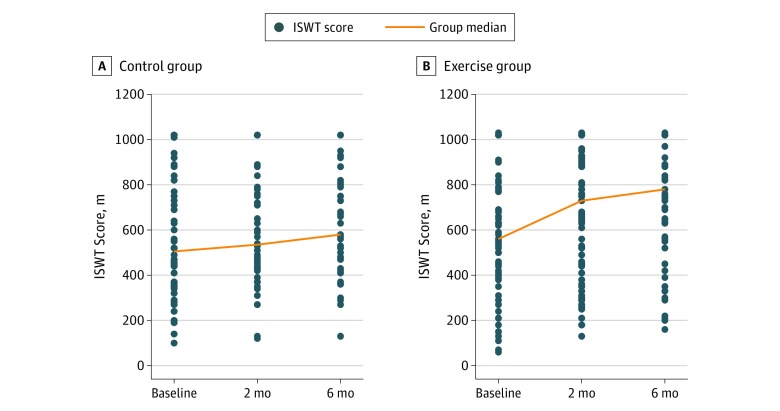
Meters Walked on the Incremental Shuttle Walk Test (ISWT) at Baseline and Follow-up

Table 2 depicts results for the primary outcome (ie, ISWT) and for the secondary outcomes (ie, PEmbQoL and EQ-5D-3L) for the period from baseline to 6-month follow-up. The EG achieved a mean (SD) improvement of 104 (106) m on the ISWT; the CG achieved a mean (SD) improvement of 78 (127) m, with a nonsignificant between-group difference of 25 m (95% CI, −20 to 70 m; *P* = .27). Figure 3 shows the changes in walking distance on the ISWT at 2 and 6 months for both groups. Results from the sensitivity analysis, which used the last observation carried forward method, reduced the mean (SD) improvement for the EG group to 95 (108) m and for the CG to 65 (114) m, with a nonsignificant between-group difference of 31.5 m (95% CI, −6.0 to 69.1 m; *P* = .10).

**Table 2.** Within-Group and Between-Group Differences From Baseline to 6 Months in the Exercise Group vs the Control Group

Outcome	Exercise Group			Control Group			Between-Group Difference, Mean (95% CI)	*P* Value^a^
	Baseline	6-mo Follow-up	Within-Group Difference, Mean (95% CI)	Baseline	6-mo Follow-up	Within-Group Difference, Mean (95% CI)		
Incremental Shuttle Walk Test, mean (SD), m	582 (285)	714 (270)	104 (75 to 132)^b^	563 (266)	637 (241)	78 (42 to 114)^b^	25 (−20 to 70)	.27
PEmbQOL score, median (IQR)^c^	30 (21 to 45)	8 (3 to 26)	−20 (−24 to −15)^b^	26 (17 to 45)	10 (2 to 20)	−17 (−22 to −11)^b^	3.0 (−3.7 to 9.7)	.39
EuroQol-5D-3L score, median (IQR)^d^	0.818 (0.77 to 1.00)	1.000 (0.824 to 1.000)	0.093 (0.061 to 0.125)^b^	0.824 (0.723 to 1.000)	1.000 (0.824 to 1.000)	0.077 (0.040 to 0.113)^b^	0.017 (−0.032 to 0.065)	.50

Abbreviation: EuroQol-5D-3L, EuroQol–5 Dimensions–3 Levels; IQR, interquartile range, PEmbQOL, Pulmonary Embolism Quality-of-Life.

^a^ *P* value calculated with Student *t* test.

^b^ *P* < .001 (paired *t* test).

^c^ Pulmonary Embolism Quality of Life has a range of 0 to 100, with a higher score indicating worse quality of life.

^d^ EuroQol-5 Dimensions has a range of −0.624 to 1.000, with 1.000 indicating perfect health.

Both groups achieved improvements on the PEmbQOL and the EQ-5D-3L, but no statistically significant differences between groups were found. The EG group had a median (IQR) improvement on the PEmbQOL of −20 (−24 to −15) points, and the CG had a median (IQR) improvement of −17 (−22 to −11) points. The EG had a median (IQR) improvement on the EQ-5D-3L of 0.093 (0.061 to 0.125) points, and the CG had a median (IQR) improvement of 0.077 (0.040 to 0.113) points. The mean difference on the PEmbQoL was 3.0 points (95% CI, −3.7 to 9.9 points; *P* = .39) and on the EQ-5D-3L, 0.017 points (95% CI, −0.032 to 0.065 points; *P* = .50) (Table 2).

In the EG, 27 patients reported sick leave days at baseline, of whom 24 (88.9%) reported fit-for-duty at the 6-month follow-up. In the CG, 18 reported sick leave at baseline, of whom 17 (94.4%) reported fit-for-duty at the 6-month follow-up, resulting in a nonsignificant risk difference of 5.5 between the groups (*P* = .49).

Most patients (64 [94.1%] in the CG and 60 [87.0%] in the EG) did not use psychotropic drugs at baseline, and this distribution had not changed at the 6-month follow-up. There were no reports of adverse events related to the interventions during the follow-up period.

### Compliance and As-Treated Analysis

Of the 69 patients allocated to the EG, 42 (60.9%) were compliant (ie, completed ≥18 exercise sessions) and 7 (10.1%) were non-compliant. The remaining 9 patients (13.0%) who had completed the 6-month follow up but did not return their exercise diaries were considered noncompliant.

A per-protocol analysis was performed, keeping only the compliant patients in the EG, with the noncompliant patients excluded from the analysis. This increased the mean improvement on the ISWT in the EG to 117.9 m (95% CI, 82.4 to 153.4 m), increasing the between-group difference to 39.5 m (95% CI, −11.3 to 90.2 m), although still without statistical significance (*P* = .13).

### Noncompleters

A total of 15 patients (10.7%) dropped out during the trial (Figure 2). To assess the potential effect of this on the study results, baseline demographic characteristics and outcome values were compared with those of the completers, showing that noncompleters had a higher PESI score compared with completers, indicating a more severe PE condition at baseline. Among noncompleters, 8 (53.3%) had a PESI of 0, 3 (20.0%) had a PESI of 1, 3 (20.0%) had a PESI of 2, and 1 (6.7%) had a PESI of 3. Among completers, 87 (71.3%) had a PESI of 0, 25 (20.5%) had a PESI of 1, 9 (7.4%) had a PESI of 2, and 0 had a PESI of 3. Furthermore, 9 patients from the CG and 4 from the EG did not perform the ISWT at 6 months but did return questionnaires. These patients did not differ from ISWT completers on age, sex, disease severity, or baseline outcome variables, although they tended to perform more poorly on baseline ISWT than the respondents (mean [SD]: 500 [314] m vs 586 [270] m).

## Discussion

Compared with nurse consultations alone, participating in 8 weeks of home-based exercise in addition to nurse consultations did not result in significantly greater improvements in terms of physical capacity and quality of life. Both groups achieved clinically relevant improvements on both primary and secondary outcomes. Moreover, we found that initiating the intervention 2 to 3 weeks after the PE event was safe and feasible and did not entail any adverse events, which is in accordance with previously published studies of early ambulation or exercise following acute PE.^17,18,19^

There are several considerations to be made when interpreting our findings. The use of an active control intervention, ie, specialized nurse consultations and tests of physical capacity, may have diluted the true effects of the rehabilitation intervention. The consultations and physical test shortly after discharge may have reassured the patients about the safety of exerting themselves physically after the PE event, thus reducing the risk of kinesophobia in both groups. Kinesophobia has been found to be a major risk factor for reduced physical activity in several patient populations, leading to negative outcomes in rehabilitation and disease trajectories.^33,34,35^ Most patients volunteering for the study had a low burden of comorbidity. This was reflected in their baseline scores on the EQ-5D-3L, which were close to Danish population norms despite their recent PE events.^36^ This is in opposition to several other prospective studies reporting significantly worse quality of life in patients with a previous PE event.^5,6,37,38,39^ However, their mean physical capacity, as measured with the ISWT, was worse than reported age-specific reference values at baseline but increased to comparable values at the 6-month follow up.^40,41^ This also leads us to consider the confounding effect of time, especially in relation to the acuteness of the PE event. The natural history of PE, given appropriate diagnosis and medication, is a fast reduction in dyspnea and chest pain shortly after initiating the medical treatment.^4^ Therefore, for future studies, considerations should be given to the timing of initiating a rehabilitation intervention and to a screening for physical and psychological risk factors 3 to 6 months after the PE event, with the aim of targeting the rehabilitation intervention to patients who have not recovered physically or mentally after an acute PE.^12,42,43^

### Strengths and Limitations

To our knowledge, the current study is the first to test a rehabilitation program in a randomized design and with a larger sample of patients with acute PE. Our study has several strengths, including randomization, the use of validated outcome measures, the use of standardized test manuals, and supervision of testing procedures during the study period to ensure similar performance of all intervention providers and outcome assessors.^24^

This study has limitations. A limitation is the choice of the ISWT as a measure of physical capacity. The test proved to have a ceiling effect in our study population, with approximately 35% of patients in both groups achieving the maximum distance of 1020 m. Furthermore, the variance (ie, SD) was higher than assumed (280 m vs 185 m). With a minimal clinically important difference of 70 m, this would have required a sample of 250 patients in each group. Therefore, for future studies, we would recommend a different test that more accurately assesses physical capacity, eg, the Watt max test.^44^

## Conclusions

In this randomized clinical trial of 140 patients with acute PE, a rehabilitation intervention entailing an 8-week home-based exercise program in addition to nurse consultations did not show significantly better outcomes in terms of physical capacity and quality of life. Importantly, all patients achieved improvements in the outcomes during the 6-month follow-up period. The current study cannot make any conclusions regarding the optimal type, content, or frequency of a rehabilitation program but adds valuable knowledge for further research in the field. First, initiating an exercise program soon after discharge was found to be feasible and without adverse events. Second, the ISWT was not an ideal measure of physical capacity in this population. Furthermore, a different recruitment method or trial design should be considered to ensure the inclusion of patients with more severe conditions of PE and/or a high comorbidity burden.

## Notes

Supplement 1. **Trial Protocol**

Supplement 2. **Data Sharing Statement**
