# Supplementary material for: Effect of a Physiotherapist-Guided Home-Based Exercise Intervention on Physical Capacity and Patient-Reported Outcomes Among Patients With Acute Pulmonary Embolism: A Randomized Clinical Trial
Source: JAMA Netw Open. 2020 Feb 28;3(2):e200064. doi: 10.1001/jamanetworkopen.2020.0064 (PMC7049077; doi:10.1001/jamanetworkopen.2020.0064)
Supplement: Supplement 1. — Trial Protocol [file jamanetwopen-3-e200064-s001.pdf]

Translation of the approved study protocol (only methods section in agreement with editor)

Methods:

Design and recruiting: The study is a randomized multicenter trial with 6 month follow-up with the participation of Regionalhospitals Silkeborg, Viborg, Horsens, Herning, and Aalborg University Hospital. A total of 140 patients with medically treated lung embolism (LE) will be included in the study, 70 in each group (Figure 1). The inclusion criteria are as follows: 1) objectively verified acute PE (ICD10 I260 and ICD10= I269); including recurrent LE; 2) treatment of LE with anticoagulant drugs; 3) 18–80 years of age; and 4) competency in the Danish language. Patients will be excluded in the case of: 1) PE as a secondary finding in relation to a scan performed due to another disease; 2) severe co-morbidity (e.g., severe heart disease, severe, active cancer, severe psychiatric disease, e.g. schizophrenia or depression). The nurses or physiotherapists at the cardiac wards of the participating hospitals will perform daily screening of patients hospitalized at the ward with the aim to recruit eligible patients according to the above criteria. On the day of discharge eligible patients are informed about the study, and the written study information is handed out (Appendix 1). Patients have a right to two days of deliberation time, and will be contacted by telephone hereafter to ask for participation in the project. By acceptance of participation written consent is retrieved (appendix 2) when the patients arrive for their baseline test in the AK-clinic (anti-coagulation clinic) app. 14 days after discharge. Following the baseline tests, patients are randomized to either the control group or the intervention group using opaque, sealed envelopes. Block randomization by hospital is used to ensure that approximately an equal number of patients at each hospital will be allocated to the control- and the intervention group, respectively.
[Figure 1. Flowchart as in manuscript]

Outcomes and methods of measurement: Data on patient's age, sex, BMI, medical treatment of LE, diagnosis (cause of LE) are retrieved from medical records at baseline.

- Primary outcome: Change in physical capacity, measured with the Incremental Shuttle Walk Test (appendix 3) from baseline to 6 months follow-up.
- Secondary outcomes: Change from baseline to 6 months follow-up on the following parameters:
    - Disease-specific quality of life measured with the Pulmonary Embolism Quality of Life questionnaire
    - Generic quality of life measured with EuroQol-5 Dimensions
    - Sick leave (number of days in the past 4 weeks)
    - Use of painkillers and psychoactive drugs the past 4 weeks.

The secondary outcome measures are collected in one questionnaire (appendix 4)
The primary endpoint is 6 months after baseline test. In addition an intermediary measurement is performed 8 weeks after baseline (after termination of exercise program, see figure 1).

The Incremental Shuttle Walk test (ISWT) has been developed for assessing and measuring changes in a person’s maximum walking capacity. The reliability and validity of the test has been shown in several studies and has been found acceptable. Studies show a reasonable association between maximal oxygen uptake and the ISWT, and that this association is better than the association between maximum oxygen uptake and the 6 minute walking test. The test has been validated within a number of study populations, including patients with heart and lung diseases [18].

The Pulmonary Embolism Quality-of-Life (PEmb-QoL) is a disease-specific quality of life questionnaire, containing six dimensions, covering frequency of complaints (8 items), activities of daily living (ADL) limitations (13 items), work-related problems (4 items), social limitations (1 item), intensity of complaints (2 items), and emotional complaints (10 items).

The EuroQol Five Dimensions (EQ-5D) is a generic quality of life questionnaire comprising both a descriptive part with 5 dimensions and the visual analogue scale EQ-VAS. The five dimensions mobility, self-care, usual activities, pain/discomfort, and anxiety/depression) [22, 23].

Sick leave and use of psychotropic drugs is gathered in questionnaires. Regarding sick leave the patient is asked to state the number of days’ sick leave within the last four weeks according to the following categories: 0 work days; 1-4 days per week; 5-7 days per week. Regarding use of psychotropic drugs (e.g antidepressive medicine, anxiety reducing medication, sleeping pills) the patient is asked to state their average weekly use within the last four weeks according to the following categories: 0 days per week, 1–4 days per week, and 5–7 days per week.

The baseline measurements are performed by the physiotherapist who sees the patient at he baseline visit 14 days after their discharge. The following tests at 8 weeks and 6 months are performed by a project physiotherapist, who is blinded as to the patient's group allocation. When the patient is summoned for the follow-up tests it is underlined that the patients must not reveal their group allocation.

Tabel 1. Overview of measurement timepoints

**Timepoint**	Baseline<br>14 days after discharge	1st follow-up<br>8 weeks after baseline	2nd follow-up<br>6 months after basleine
**Outcome**			
Incr SWT	X	X	X
PEmb-QoL	X	X	X
EQ-5D	X	X	X
Sick leave	X	X	X
Medication	X	X	X

Incr SWT = Incremental Shuttle Walk Test; PEmb-QoL = Pulmonary Embolism Quality of Life Questionnaire; EQ-5D = EuroQol 5 Dimensions

Interventions

Group 1, usual care: A usual course of treatment typically entails 3-5 days of hospitalization where anticoagulant treatment is initiated. The patient and his or her relatives receive general information about the disease and the course of treatment, the medication, and future prevention of embolism. Approximately 2 weeks after discharge the patient is seen at the hospital for control of their medication and the following outpatient visits are planned individually depending on the patient's needs. Any other treatment that may be offered at the hospitals, e.g. contact with physiotherapists during hospitalization can vary between hospitals. The patients are scheduled for physical tests and completion of questionnaires at 8 weeks and 6 monts.

Group 2, homebased exercise: In addition to usual care, patients are offered participation in an eight-week home-based exercise program with telephone follow-ups after 1, 2 and 4 weeks. Following the completion of the baseline measurements the physiotherapist has a dialogue with the patient regarding the importance of exercise and how the patient can schedule the exercise in their everyday life. In brief, the patients are recommended to exercise for a minimum of three times per week for 1 hour, with intervals (20 minutes in total) at a high intensity level, where only very short sentences of speech are possible). The remaining 40 minutes are at a moderate intensity, where you are only slightly out of breath, but having a conversation is possible. The patients can choose whatever type of exercise they prefer, and they are generally encouraged to choose an exercise modality they already perform, or a modality they have previously had positive experiences with. The patients record all their training sessions in a diary handed out at the first visit (appendix 5). The training diary can be handed out in paper or be sent on email. The training diary will be used for the motivational follow-up calls made by the physiotherapist at 1, 2 and 4 weeks. After the 8 weeks program is finished, the patient brings the diary to the hospital at the subsequent follow-up test. Patients in the intervention group are scheduled for physical tests and completion of questionnaires at 8 weeks and 6 months like the control group.

Analysis and evaluation: For the primary outcome, ISWT, parametrical statistics are used to describe and analyse data, if data are normally distributed. For the secondary outcomes, PEmbQoL and EQ-5D, data are on an ordinal scale, and hence data are described and analyzed using non-parametric statistics. Sick leave (number of days) are described and analyzed using parametrical statistics if data are normally distributed. For the evaluation of psychoactive drugs the chi2 test is used. The power calculation is based on the ISWT. The smallest clinical relevant difference has been reported to be between 50 m. (COPD rehabilitation) and 70 m. (cardiac rehabilitation). As a clinically relevant difference has not been investigated for LE patients and arbitrarily clinically relevant difference of 60 meters was decided upon. With a power of 80% and a significance level of 5% a sample size of 60 patients is needed for each group. With an expected drop-out rate of 10% in each group we aim to include 140 patients in total.

Regionshospitalet Silkeborg ● Diagnostisk Center ● Falkevej 1-3 ● 8600 Silkeborg

Effekten af et 8-ugers træningsforløb på fysisk kapacitet og livskvalitet hos patienter med lunge-emboli. En klinisk randomiseret undersøgelse

Projektgruppe:
Pernille Ravn, fysioterapeut
Hanne Mikkelsen, sygeplejerske
Lars Frost, overlæge, dr. med.
Mette Krag Svendsen, ledende terapeut
Nanna Rolving, forskningsansvarlig terapeut, ph.d.

Baggrund:

I Danmark findes der på nuværende tidspunkt ikke noget rehabiliteringstilbud til de ca. 2.500-3.000 mennesker der årligt rammes af en blodprop i lungen, også kaldet lunge-emboli (LE) [1]. Patienterne følges efter indlæggelsen således kun medicineringsmæssigt af en sygeplejerske og læge, men tilbydes som udgangspunkt ikke yderligere rehabiliterende tiltag, som det eksempelvis gælder for hjerte- eller KOL patienter. Patienterne betragtes fra et medicinsk synspunkt som raske når den antikoagulerende behandling iværksat og er velreguleret, men der en række patientoplevede konsekvenser af sygdommen, der ikke tages hånd om i det nuværende behandlingstilbud. Det opleves i praksis at patienterne har mange spørgsmål og bekymringer vedrørende fysiske, psykiske og sociale elementer fx Hvad må jeg fysisk? Skal jeg være bange for at jeg får en lunge-emboli igen? Hvornår må jeg begynde på arbejde?

Den foreliggende viden på området fokuserer primært på diagnostik og lægelig behandling af tilstanden, samt den kortsigtede prognose ift. mortalitet og komplikationer [2]. Der er meget begrænset viden om hvordan LE påvirker patienternes hverdagsliv efterfølgende, og kun fire studier har belyst patientrapporterede outcomes [3-6]. I to af studierne fandt man, at mellem 20 % og 40 % af patienterne oplevede fysiske begrænsninger, åndenød og nedsat gangdistance 6 mdr. efter behandling for LE [5, 6]. I det tredje studie rapporterede 44 % af patienterne markant dårligere livskvalitet sammenlignet med baggrundspopulationen 3½ år efter deres LE [3]. Derudover har en dansk registerundersøgelse påvist, at yngre patienter der får LE (< 33 år) har et signifikant øget forbrug af psykofarmaka op til 5 år efter [4].

Hvorvidt en indsats med fysisk rehabilitering til denne målgruppe kan forbedre behandlingsresultatet efter LE på patientrelaterede outcomes er uvist, da der ikke foreligger studier der har undersøgt dette. Der er fundet to studier af lavere kvalitet (et randomiseret pilotprojekt (n=39) samt et prospektivt kohorte studie (n=35)), der har forsøgt at belyse effekten af en træningsindsats til henholdsvis patienter med post-trombotisk syndrom efter dyb venetrombose, samt patienter med kronisk pulmonal hypertension [7, 8]. Begge studier fandt en god effekt af træningsindsatsen i forhold til livskvalitet, symptomer på post-trombotisk syndrom samt fysisk arbejdskapacitet. Ser man på mere velundersøgte områder som hjerte- og KOL-rehabilitering, foreligger der solid evidens for, at fysisk træning har en god effekt på bl.a. livskvalitet, fysisk kapacitet, træthed og åndenød [9, 10].

Formålet med undersøgelsen er derfor at undersøge effekten af et forløb med 8-ugers hjemmetræning på fysisk formåen, livskvalitet, sygefravær og forbrug af psykofarmaka efter medicinsk behandling for LE.

Metode:

Design og rekruttering: Undersøgelsen er et randomiseret multicenter-studie med 6 måneders opfølgning, med deltagelse af Regionshospitalerne Silkeborg, Viborg, Randers, Horsens og Herning. Det er planlagt at i alt 140 patienter med medicinsk behandlet LE skal indgå i studiet, 70 i hver gruppe (Figur 1). Inklusionskriterierne er som følger: 1) Objektivt verificeret akut lunge-emboli (ICD10 I260 og ICD10 I269), inklusiv recidiv 2) Behandling med antikoagulerende medicin; 3) 18-70 år; 4) Taler og forstår dansk. Patienterne ekskluderes ved 1) Lunge-emboli som bi-fund ved scanning foretaget pga. anden sygdom; 2) Svær co-morbiditet (fx svær hjertesygdom, svær kronisk obstruktiv lungelidelse, cancer, svær psykiatrisk sygdom som fx skizofreni eller depression). Sygeplejersker eller fysioterapeuter i hjerteteamet på det enkelte sygehus udfører dagligt screeningsprocedure i afdelingen med henblik på at rekruttere egnede patienter i henhold til ovenstående kriterier. På dagen for udskrivelse informeres egnede patienter om den i gangværende undersøgelse, og den skriftlige deltagerinformation udleveres (Bilag 1). Patienterne har ret til to dages betænkningstid, og de kontaktes herefter telefonisk med anmodning om deltagelse i projektet. Ved accept af deltagelse indhentes skriftligt samtykke (Bilag 2) i forbindelse med patientens baseline test ved deres første besøg i AK-klinikken ca. 14 dage efter udskrivelsen. Efter baseline testen er udført, randomiseres patienterne til kontrol- eller forsøgsgruppe ved hjælp af uigennemsigtige kuverter. Der anvendes blokrandomisering på hospitalsbasis, for at sikre at ca. lige mange patienter på hvert hospital randomiseres til henholdsvis kontrol- og forsøgsgruppe

Figur 1. Flowdiagram.

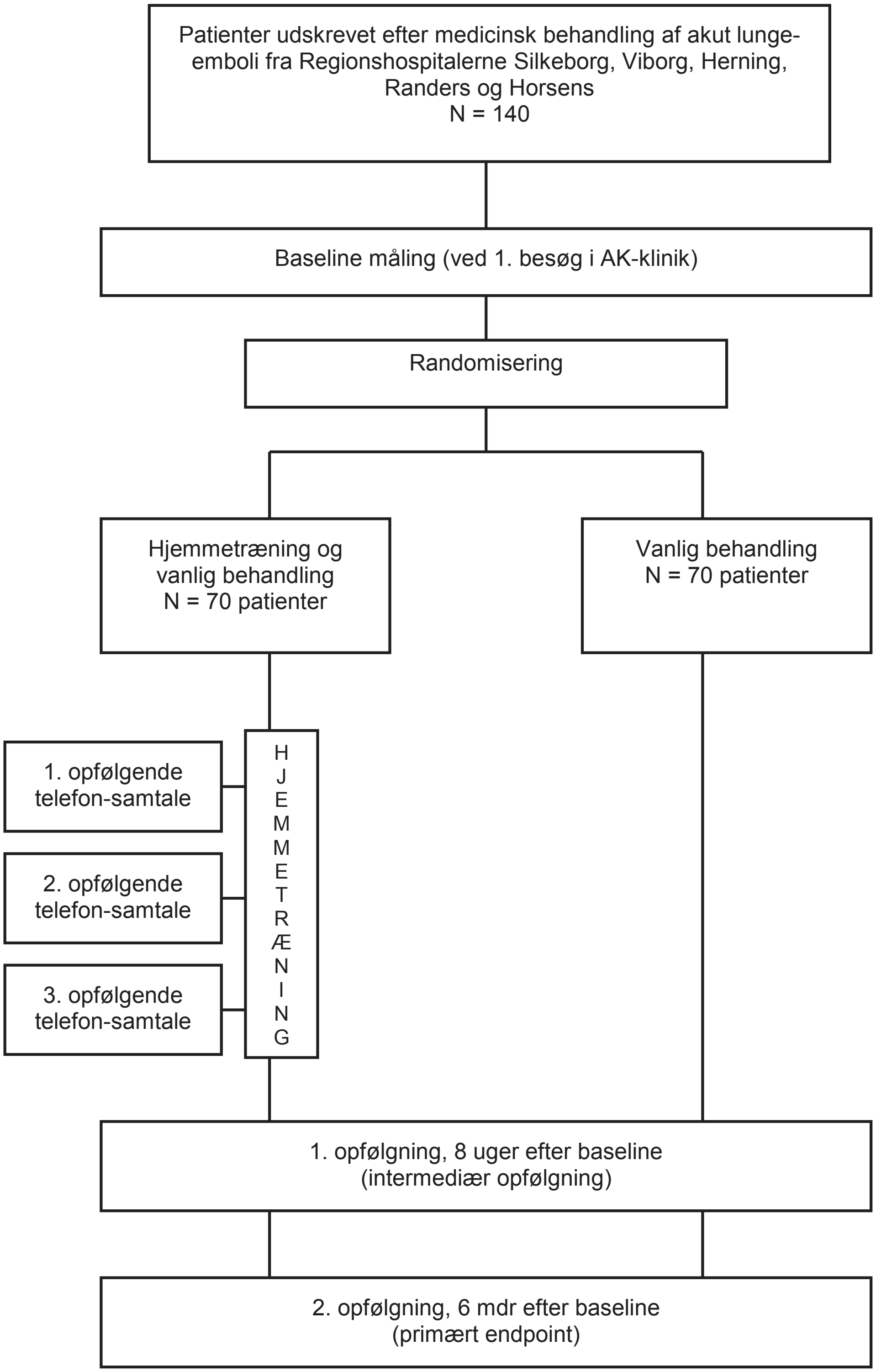

Effektmål og målemetoder: Der videregives oplysninger fra patientens journal om alder, køn, BMI, medicinsk behandling af LE og diagnose (årsag til LE) ved baseline.

- Primære effektmål: Ændring i fysisk kapacitet, målt med Incremental Shuttle Walk Test (Bilag 3), fra basline til 6 måneders follow-up.
- Sekundære effektmål: Ændring fra baseline til 6 måneders follow-up på følgende parametre:
  - Sygdomsspecifik livskvalitet målt med Pulmonary Embolism Quality-of-Life questionnaire
  - Generisk livskvalitet malt med EuroQol-5 Dimensions
  - Sygefravær (antal dage i de sidste 4 uger)
  - Forbrug af smertestillende medicin og psykofarmaka (i de sidste 4 uger).

De sekundære effektmål er samlet i ét spørgeskema (Bilag 4).
Det primære endpoint er 6 måneder efter baseline måling. Herudover foretages også en intermediær måling 8 uger efter baseline (efter endt træningsforløb, se Figur 1).

Incremental Shuttle Walk Test (ISWT) er udviklet med henblik på at vurdere og måle ændringer i maksimal gangkapacitet [11]. Testens reliabilitet og validitet er belyst i flere studier og er generelt fundet acceptabel. Studier viser, at der er fornuftig sammenhæng mellem maximal iltoptagelse og ISWT, og denne sammenhæng er bedre end sammenhængen mellem maximal iltoptagelse og eksempelvis 6 minutters gangtest. Testen er valideret i en lang række sygdomspopulationer, herunder hos patienter med lunge- og hjerte/kar sygdomme [12].
Pulmonary Emboli Quality-of-Life (PEmb-QoL) er et sygdomsspecifikt livskvalitetsspørgeskema bestående af 9 overordnede dimensioner, der dækker emnerne *hyppighed af symptomer* (8 spørgsmål), *begrænsninger i dagligdags aktiviteter* (13 items), *arbejdsrelaterede spørgsmål* (4 items), *sociale begrænsninger* (1 item), *intensitet af symptomer* (2 items) og *emotionelle symptomer* (10 items)[13-15].
EuroQol 5 Dimensions (EQ5D) er et generisk livskvalitetsspørgeskema, bestående dels af en deskriptiv del med 5 dimensioner samt den visuelle analog skala EQ-VAS. De 5 dimensioner dækker over mobilitet, selvhjulpenhed, sædvanlige aktiviteter, smerter/ubehag og angst/depression [16, 17].

Sygefravær og forbrug af psykofarmaka indhentes ved brug af spørgeskema. For sygefravær bedes patienten angive deres samlede sygefravær indenfor de sidste 4 uger i følgende kategorier 0 dage; <5 arbejdsdage; <10 arbejdsdage; ≥10 arbejdsdage. For forbrug af psykofarmaka (fx antidepressiva, angstdæmpende medicin, sovemedicin) bedes patient angive deres ugentlige forbrug i de sidste 4 uger i kategorierne 0 dage, 1-4 dage per uge, 5-7 dage per uge.

Baselinemålingerne udføres af den fysioterapeut, der ser patienten i ambulatoriet, ca. 14 dage efter deres udskrivelse. De efterfølgende tests ved 8 uger og 6 måneder udføres af en projektfysioterapeut, der er blindet for, om patienten er i forsøgs- eller kontrolgruppen. Ved indkaldelse til opfølgende tests indskærpes det derfor overfor patienten, at de *ikke* må afsløre overfor den testende fysioterapeut, hvilken gruppe de har været i.

Tabel 1. Overblik over måletidspunkter:

**Måletidspunkt**	**Baseline:** 14. dage efter udskrivelse	**1. opfølgning:** 8 uger efter baseline	**2. opfølgning:** 6 mdr. efter baseline
**Effektmål**			
Incr SWT	X	X	X
Pemb-QoL	X	X	X
EQ-5D	X	X	X
Sygefravær	X	X	X
Medicin	X	X	X

Incr SWT = Incremental Shuttle Walk test; Pemb-QoL = Pulmonary Embolism Quality of Life Questionnaire; EQ-5D = EuroQoL 5 dimensions

Interventioner:

Gruppe 1, vanlig behandling: Et vanligt behandlingsforløb indebærer typisk 3-5 dages indlæggelse hvor antikoagulerende behandling opstartes. Der gives generel information til patient og pårørende om sygdomsforløbet, den medicinske behandling og forebyggelse fremadrettet. Cirka 2 uger efter udskrivelse indkaldes patienten herefter til kontrol af deres antikoagulerende medicin, og efterfølgende kontroller aftales individuelt efter behov. Øvrig behandling der tilbydes, fx kontakt til fysioterapeut under indlæggelse, kan variere mellem hospitalerne. Patienterne indkaldes til fysiske tests og udfyldelse af spørgeskema ved 8 uger og 6 mdr.

Gruppe 2, hjemmetræning: Udover vanlig behandling tilbydes patienterne at deltage i et 8 ugers hjemmetræningsforløb med opfølgende telefonkontakt efter 1 uge, 2 uger og 4 uger. Efter gennemførelsen af baseline målingen har fysioterapeuten en samtale med patienten omkring

vigtigheden af træning og hvordan patienten bedst kan tilrettelægge træningen i sin hverdag. Kort fortalt opfordres patienten til at træne minimum 3 gange om ugen af én times varighed, med indlagte intervaller (i alt 20 min) på intensivt niveau, svarende til at man kun tale i korte sætninger. De resterende 40 min er i moderat intensivt, hvor man bliver lettere forpustet, men hvor samtale er mulig. Patienterne må selv vælge træningsformen, og de opfordres til at vælge en træningsform de dyrker i forvejen eller har positive erfaringer med. Patienterne skal registrere alle deres træningspas i en træningsdagbog (Bilag 5). Træningsdagbogen kan patienten enten få udleveret i papirform eller få tilsendt per e-mail. Træningsdagbogen bruges som udgangspunkt for de motiverende opfølgningssamtaler (telefonisk), som fysioterapeuten foretager efter hhv. 1, 2 og 4 ugers træning. Efter de 8 ugers træning er afsluttet medbringer patienten træningsdagbøgerne til deres første opfølgende test på hospitalet. Er dagbøgerne udfyldt elektronisk indsendes de per mail. Patienterne indkaldes til fysiske tests og udfyldelse af spørgeskema ved 8 uger og 6 mdr. ligesom kontrol gruppen.

Analyse og evaluering: For det primære outcome, ISWT, anvendes parametrisk statistik til at beskrive og analysere data, såfremt data er normalt-fordelte. For de sekundære outcome, PEmb-QoL og EQ-5D, er der tale om ordinale data, og data beskrives og analyseres derfor med non-parametrisk statistik. Sygefravær (antal dage) beskrives og analyseres ved hjælp af parametrisk statistik, såfremt data er normalt-fordelt. Til evaluering af forbrug af psykofarmaka anvendes chi2 test. Power beregningen baseres på det ISWT. Den mindste klinisk relevante forskel er angivet til at være mellem 50 m. (KOL-rehabilitering) og 70 m. (hjerterehabilitering). Da en klinisk relevant forskel ikke er undersøgt for LE patienter valgtes en arbitrært sat klinisk relevant forskel på 60 m. Med en power på 80 % og signifikansniveauet sat til 5 % kræves en stikprøvestørrelse på 60 deltagere i hver gruppe. Med et forventet frafald på 10 % i hver gruppe sigtes der således mod at inkludere i alt 140 patienter.

Etik: Patienterne informeres mundtligt og skriftligt om undersøgelsens formål, og der indhentes skriftligt samtykke hos de deltagende patienter til at anvende deres data. Patienterne informeres om, at det er frivilligt at deltage, og at de til enhver tid har ret til at trække deres samtykke tilbage. Studiet er anmeldt til Region Midts paraplyanmeldelse til Datatilsynet den 26/08/2015 (godkendelse afventes), samt til Videnskabs Etisk Komité for Region Midtjylland (godkendelse afventes).

Oplysning om økonomisk støtte og organisering:

Projektet er initieret af overlæge Lars Frost, fysioterapeut Pernille Ravn og sygeplejerske Hanne Mikkelsen, Diagnostisk Center, Regionshospitalet Silkeborg, og med yderligere deltagelse af de kardiologiske afdelinger og fysioterapi- og ergoterapiafdelingerne ved regionshospitalerne Viborg, Randers, Horsens og Herning. Styregruppen for projektet består af overlæge Lars Frost, forskningsansvarlig terapeut Nanna Rolving, ledende terapeut Mette Krag Svendsen og fysioterapeut Pernille Ravn.

Der er endnu ikke indhentet økonomisk støtte til projektet. Aflønning af projektgruppens medlemmer (Lars Frost, Pernille Ravn, Hanne Mikkelsen, Nanna Rolving og Mette Krag Svendsen) til det forberedende arbejde der har pågået indtil nu, har været afholdt af Diagnostisk Center, Regionshospitalet Silkeborg. Der søges midler til finansiering af VIP-midler, TAP-løn, drift og formidling hos Region Midtjyllands Sundhedsvidenskabelige Forskningsfond, Hjerteforeningen, Danske Fysioterapeuters Forskningsfond, Lundbeck fonden og Augustinusfonden. Indhentede fondsmidler vil blive indsat på en øremærket projektkonto i HE Midt regi (oprettes af økonomi-afdelingen). De forsøgsansvarlige, Lars Frost og Nanna Rolving, har ingen tilknytning til nævnte fonde.

Tidsperspektiv for projektet:

Juni – august 2015: færdiggørelse af testprotokol og informationsmateriale til patienter

August-september 2015: pilottest på Regionshospitalet Silkeborg

August 2015: Anmeldelse af projekt til Videnskabsetisk Komité og datatilsynet samt fondsansøgninger

Oktober - november 2015: Planlægning- og undervisningsmøder med terapeuter (og øvrigt relevant personale) på de deltagende hospitaler, afprøvning af hjemmetræning og testprotokoller.

December 2015: Fælles opstartsmøde for alle projektarbejdere på deltagende hospitaler (heldagsmøde)

Januar 2016: Projektet kører i gang. Inklusionen af patienter fortsætter indtil der er ca. 140 patienter inkluderet i alt. Dette forventes at tage 1-1½ år.

Januar 2016 – juni 2017: Der afholdes løbende supervisionsmøder på de deltagende hospitaler for at sikre fremdrift og ensartet udførelse på de 5 matrikler.

December 2017: Efter 6 måneders opfølgningen er komplet for alle patienter påbegyndes data-analyse og formidling / publicering.

Formidling

Der stiles mod publicering af projektets resultater i både nationale og internationale tidsskrifter indenfor rehabilitering og kardiologi, ligesom projektets resultater vil blive præsenteret ved nationale og internationale konferencer. Positive, negative såvel som inkonklusive resultater vil blive offentliggjort.

Anvendelse / perspektiv: Vi forventer, at den afprøvede indsats vil forbedre de samlede behandlingsresultater, for patienter der får en blodprop i lungen. Projektet kan således bidrage med ny viden om rehabilitering af patientgruppen, og kan danne grundlag for nye anbefalinger på området. På grund af den meget sparsomme viden om patientoplevede konsekvenser af sygdommen (fx livskvalitet, sygefravær, angst, fysisk funktionsevne mv.) for denne målgruppe, kan projektet også være med til at generere ny og vigtig viden herom.

Litteraturliste

1. Hjerteforeningen (2015) Hjertetal. Lungeemboli, nye tilfælde, 2012. 2015

2. Kahn SR, Houweling AH, Granton J, Rudski L, Dennie C, Hirsch A (2014) Long-term outcomes after pulmonary embolism: current knowledge and future research. Blood Coagul Fibrinolysis DOI 10.1097/MBC.0000000000000070 [doi]

3. Klok FA, van Kralingen KW, van Dijk AP, Heyning FH, Vliegen HW, Kaptein AA, Huisman MV (2010) Quality of life in long-term survivors of acute pulmonary embolism. Chest DOI 10.1378/chest.09-2482 [doi]

4. Hojen AA, Gorst-Rasmussen A, Lip GY, Lane DA, Rasmussen LH, Sorensen EE, Larsen TB (2015) Use of psychotropic drugs following venous thromboembolism in youth. A nationwide cohort study. Thromb Res DOI 10.1016/j.thromres.2015.01.024 [doi]

5. Kline JA, Steuerwald MT, Marchick MR, Hernandez-Nino J, Rose GA (2009) Prospective evaluation of right ventricular function and functional status 6 months after acute submassive pulmonary embolism: frequency of persistent or subsequent elevation in estimated pulmonary artery pressure. Chest DOI 10.1378/chest.08-2988 [doi]

6. Stevinson BG, Hernandez-Nino J, Rose G, Kline JA (2007) Echocardiographic and functional cardiopulmonary problems 6 months after first-time pulmonary embolism in previously healthy patients. Eur Heart J DOI ehm295 [pii]

7. Kahn SR, Shrier I, Shapiro S, Houweling AH, Hirsch AM, Reid RD, Kearon C, Rabhi K, Rodger MA, Kovacs MJ, Anderson DR, Wells PS (2011) Six-month exercise training program to treat post-thrombotic syndrome: a randomized controlled two-centre trial. CMAJ DOI 10.1503/cmaj.100248 [doi]

8. Nagel C, Prange F, Guth S, Herb J, Ehlken N, Fischer C, Reichenberger F, Rosenkranz S, Seyfarth HJ, Mayer E, Halank M, Grunig E (2012) Exercise training improves exercise capacity and quality of life in patients with inoperable or residual chronic thromboembolic pulmonary hypertension. PLoS One DOI 10.1371/journal.pone.0041603 [doi]

9. Anderson L, Taylor RS (2014) Cardiac rehabilitation for people with heart disease: an overview of Cochrane systematic reviews. Cochrane Database Syst Rev DOI 10.1002/14651858.CD011273.pub2 [doi]

10. McCarthy B, Casey D, Devane D, Murphy K, Murphy E, Lacasse Y (2015) Pulmonary rehabilitation for chronic obstructive pulmonary disease. Cochrane Database Syst Rev DOI 10.1002/14651858.CD003793.pub3 [doi]

11. Singh SJ, Morgan MD, Scott S, Walters D, Hardman AE (1992) Development of a shuttle walking test of disability in patients with chronic airways obstruction. Thorax

12. Parreira VF, Janaudis-Ferreira T, Evans RA, Mathur S, Goldstein RS, Brooks D (2014) Measurement properties of the incremental shuttle walk test. a systematic review. Chest DOI 1809990 [pii]

13. Cohn DM, Nelis EA, Busweiler LA, Kaptein AA, Middeldorp S (2009) Quality of life after pulmonary embolism: the development of the PEmb-QoL questionnaire. J Thromb Haemost DOI 10.1111/j.1538-7836.2009.03341.x [doi]

14. Klok FA, Cohn DM, Middeldorp S, Scharloo M, Buller HR, van Kralingen KW, Kaptein AA, Huisman MV (2010) Quality of life after pulmonary embolism: validation of the PEmb-QoL Questionnaire. J Thromb Haemost DOI 10.1111/j.1538-7836.2009.03726.x [doi]

15. Tavoly M, Jelsness-Jorgensen LP, Wik HS, Roaldsnes C, Sandset PM, Ghanima W (2015) Quality of life after pulmonary embolism: first cross-cultural evaluation of the pulmonary embolism quality-of-life (PEmb-QoL) questionnaire in a Norwegian cohort. Qual Life Res DOI 10.1007/s11136-014-0779-4 [doi]

16. Anonymous (1990) EuroQol - a new facility for the measurement of health-related quality of life. Health Policy

17. Wittrup-Jensen KU, Lauridsen J, Gudex C, Pedersen KM (2009) Generation of a Danish TTO value set for EQ-5D health states. Scand J Public Health
