# Supplementary material for: Effect of a Physiotherapist-Guided Home-Based Exercise Intervention on Physical Capacity and Patient-Reported Outcomes Among Patients With Acute Pulmonary Embolism: A Randomized Clinical Trial
Source: JAMA Netw Open. 2020 Feb 28;3(2):e200064. doi: 10.1001/jamanetworkopen.2020.0064 (PMC7049077; doi:10.1001/jamanetworkopen.2020.0064)
Supplement: Supplement 2. — Data Sharing Statement [file jamanetwopen-3-e200064-s002.pdf]

Data Sharing Statement

Rolving. Effect of a Physiotherapist-Guided Home-Based Exercise Intervention on Physical Capacity and Patient-Reported Outcomes Among Patients With Acute Pulmonary Embolism. *JAMA Netw Open*. Published February 28, 2020. 10.1001/jamanetworkopen.2020.0064

Data
Data available: No

Additional Information
Explanation for why data not available: Due to the European General Data Protection Regulation (GDPR) we will not make data available.
